# Obesity, Air Pollution, and Epigenetic Modifications as Risk Factors for Asthma Phenotypes

**DOI:** 10.3390/ijms27104350

**Published:** 2026-05-13

**Authors:** Velia Malizia, Angela Marina Montalbano, Anna Bonomolo, Pietro Alfano, Filippo Sapienza, Ilaria Stanisci, Stefania La Grutta, Mirella Profita

**Affiliations:** 1 Institute of Translational Pharmacology—National Research Council of Italy (IFT-CNR), Section of Palermo, 90146 Palermo, Italy; velia.malizia@ift.cnr.it (V.M.); angelamarina.montalbano@cnr.it (A.M.M.); anna.bonomolo@cnr.it (A.B.); pietro.alfano@cnr.it (P.A.); filippo.sapienza01@community.unipa.it (F.S.); stefania.lagrutta@cnr.it (S.L.G.); 2 Institute of Clinical Physiology (IFC), National Research Council (CNR), 56124 Pisa, Italy; ilariastanisci@cnr.it

**Keywords:** obesity, air pollution, epigenetic changes, asthma phenotypes

## Abstract

Multiple interacting risk factors can influence the origin of asthma. Asthma is characterized by different clinical phenotypes, each of which includes different endotypes. There are four main clinical asthma phenotypes: (1) early-onset mild allergic asthma; (2) early-onset allergic moderate-to-severe remodeled asthma; (3) late-onset non-allergic eosinophilic asthma; and (4) late-onset non-eosinophilic non-allergic asthma. The main endotypes of asthma are T-helper (Th)-2 low and Th-2 high. The identification of asthma endotypes might help precision-based care move toward the personalized management of airway inflammation. In this scenario, it is important to know how the risk factors affect the pathophysiology of asthma. Accordingly, we focus our attention on the impact of obesity and air pollutants and how these risk factors together with epigenetic alterations influence the asthma phenotype/endotype and the pathogenesis of airway diseases. Our aim is to disseminate the progress of studies in this area by reporting recent observations on the topic. Finally, we believe that data/observations enclosed in this review suggest the need of further epidemiological studies to be useful to examine simultaneously the effect of more than one risk factor on clinical and biologic parameters of asthma.

## 1. Introduction

Over the past decades, the incidence of morbidity and mortality of asthma has increased worldwide, and the prevalence of asthma places a high socio-economic burden on the population (patients and families) [1]. Asthma is a chronic inflammatory disease of the lung with reversible airflow obstruction. It is characterized by increased levels of eosinophils, airway hyperresponsiveness and remodeling with mucus hypersecretion, goblet cell metaplasia, airway inflammation, and increased levels of IgE [1]. In asthma airway inflammation is a hallmark feature. The persistence of inflammation affects oxidative stress and antioxidant defenses to generate tissue damage and remodeling. However airway inflammation and remodeling are, for the most part, independent mechanisms of asthmatic disease [2,3]. The symptoms of asthma, such as wheezing, shortness of breath, cough, chest tightness, and varying expiratory airflow limitation, are different in relation to the heterogeneity of chronic inflammation of the airways [4,5].

The onset of the pathophysiological processes of asthma originates during the early stages of life [6]. It is a disease phenotypically heterogeneous in terms of clinical (age at onset, degree of severity and response to the treatment), biological and immunological features of inflammatory mechanisms [2]. The definition of asthma phenotype is based on clinical and functional features of patients, regard multiple interacting molecular mechanisms (“molecular phenotype”). A single phenotype with the same clinical and molecular characteristics or response to the treatment is defined as an “endotype”. Patients with the same phenotype of asthma have different ‘endotypes’ [4]. Currently, the heterogeneity of asthma is defined by lung function, allergies and inflammatory pathways and not only the presence or absence of eosinophils as essential biomarkers of disease [5,7]. However, to date the definition of the individual phenotypes of asthma is not very clear; therefore, further studies and knowledge need to be developed.

Phenotypes and endotypes of asthma are affected by comorbidities, environmental stimuli and genetic contributors, including pathogens, allergens, and pollutants. These factors induce dynamic modifications in molecular pathways influencing the life of patients [5,8]. Machine learning and data analysis studies might contribute to defining a more accurate identification of similar clusters of phenotypes and endotypes of asthma [9,10,11].

One Health data integration is an interdisciplinary strategy with a focal point on human, animal, and environmental health interconnections including laboratory diagnostic and epidemiological data with environmental and biodiversity data. It defines a more accurate picture of the events involved in the disease, and might be useful to identify the right tools for asthma prevention, control, or management [12,13]. Today, integrated approaches exist that foster the framework structures of One Health data integration. In this way, specific considerations are raised, supported by operational data integration, that provide the identification of new hypotheses, insights, and One Health integrated solutions [14].

In this review, we focused on certain asthma risk factors, such as obesity, air pollution, and epigenetic modifications, with the aim of understanding some aspects of the etiology and pathophysiology of asthma phenotypes. In this context, we did not discuss the therapeutic aspects in depth, as they are more closely associated with a distinct clinical field and are addressed more specifically in asthma management guidelines and reviews.

## 2. Asthma Phenotypes/Endotype

The endotypes of asthma are T-helper (Th)-2-low and Th-2-high endotypes and sixty percent of cases are represented by the Th-2-high endotype [10,11,12]. Th2-low asthma does not share the characteristics of Th2-high asthma. Th2-low asthma is poorly responsive to corticosteroids and is not well characterized as Th2-high asthma. The absence of Th2-high inflammatory markers defines Th2-low asthma. Low levels of Th-2 inflammatory biomarkers are associated with neutrophils as dominant inflammatory cell types in asthma [5,8]. It is possible to describe three types of Th2-low asthma showing: (1) neutrophilia with low eosinophilia; (2) neutrophilia and eosinophilia, and (3) paucigranulocytic (low neutrophilia and eosinophilia). Patients with neutrophilic phenotype 1 and 2 more often have higher severity and rates of exacerbations [15]. The Th2-low endotype may be involved in Th-1 or Th-17 pathways resulting in a neutrophilic or paucigranulocytic inflammatory response (i.e., normal sputum levels of eosinophils and neutrophils) with a lack of response to corticosteroid therapy. Th2-low asthma has been described more frequently in adults than in children and comprises neutrophilic and paucigranulocytic asthma [8,16,17,18]. Th-1 and Th-3 immune pathways play main roles in the development of Th-2-low phenotypes. The Th-1 response is triggered to counteract viral infections activating TLR on epithelial cells and by the release of IL-12 from DC. IL-12 is associated with differentiation of Th-1 to produce IFN-γ in severe asthma. Th-3 immunity is mediated by Th-17 lymphocytes and IL-C3 (innate lymphoid cells type 3), which secrete cytokines belonging to the IL-17 family (IL-17 A–F and IL-22) and contribute to steroid resistance, neutrophil recruitment to the airways, mucous cell metaplasia, airway smooth muscle mass hyperplasia and fibroblast proliferation [19].

Recent studies show the efficacy of new pharmacological approaches for the treatment of asthma by using biological drugs [20]. The immunological pathways involved in clinical phenotypes describe specific biomarkers and define severe asthma endotypes (biological mechanisms), necessary to identify or predict biologic therapy which are poorly understood [21]. The identification of specific asthma endotypes might help precision-based care move toward the personalized management of inflammation. The selection of biologic drugs for the treatment of severe asthma exacerbations requires careful evaluation of the involved biomarkers such as blood eosinophils, total and specific immunoglobulin E/allergic sensitization, and fraction of exhaled nitric oxide (FeNO). Pediatric asthma exacerbations requiring intensive care admission may constitute a high-risk phenotype that is part of a high-risk subgroup, difficult to control, with frequent exacerbations and treatment resistance [22].

Novel treatments move towards targeting upstream pathways of asthma pathogenesis, coordinated by cytokines and alarmins as biological therapies effective for the adjunctive treatment of Th-2 asthma. These treatments aim to control the key proinflammatory mediators that orchestrate the activation of complex cellular networks of both innate and adaptive immune responses. Clinical traits contribute to defining the immunological profile of asthma phenotypes and how biologic treatment affects the resolution of symptoms. It has been observed that the response to anti-IL-5/anti-IL-5R therapy varies across phenotypes of high-eosinophilic Th2 asthma: patients with late-onset disease generally show a reduction in symptoms, whereas other subgroups exhibit a more limited response or are relatively refractory to biologic treatments [23]. In addition, the anti-TSLP monoclonal antibody may also be useful for patients with poorly controlled severe asthma of the Th2-low type [20]. However, the variability of asthma phenotypes underlines the need to study possible genetic alterations to carry out more in-depth research in asthma, useful for the identification of targeted pharmacological treatments [17].

**Figure 1:**
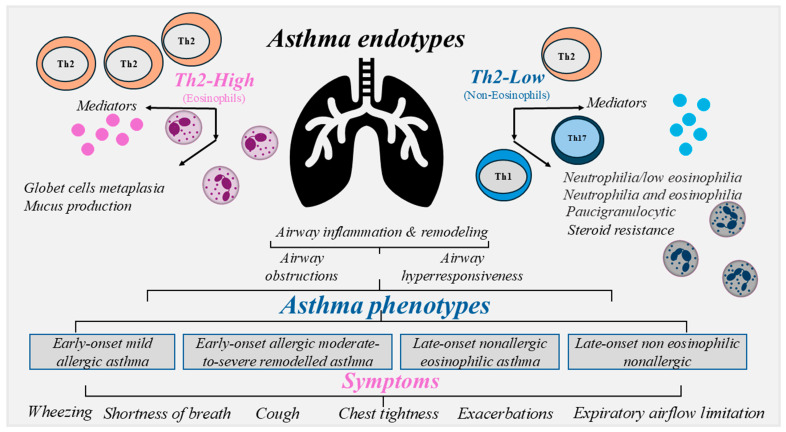
Asthma is a chronic inflammatory disease of the airways. External stimuli affect the release of mediators, mucus hypersecretion, eosinophils, high levels of IgE, goblet cell metaplasia, promoting airway inflammation and remodeling. The endotypes of asthma are Th-2-low and Th-2-high endotypes. There are three types of Th2-low asthma: (1) neutrophilia with low eosinophilia; (2) neutrophilia and eosinophilia, and (3) paucigranulocytic (low neutrophilia and eosinophilia). The Th2-low endotype may be involved in the Th-1 or Th-17 pathway resulting often in a missed response to corticosteroid. Airway inflammation and remodeling affect airway obstruction and hyperresponsiveness. There are 4 main clinical asthma phenotypes: (1) early-onset mild allergic asthma, (2) early-onset allergic moderate-to-severe remodeled asthma, (3) late-onset non-allergic eosinophilic asthma, and (4) late-onset non-eosinophilic non-allergic asthma. Consequently, the origin of the symptoms of asthma, wheezing, shortness of breath, cough, chest tightness, exacerbations and varying expiratory airflow limitation, can be different in relationship to the heterogeneity of chronic inflammation of the airways.

The heterogeneity of asthma phenotypes is often characterized by structural changes known as airway remodeling. It involves structural alterations of airway epithelial and subepithelial layers, including epithelial desquamation, thickening of the basement membrane, increased vascularity, and smooth muscle hyperplasia and hypertrophy. There are four main clinical asthma phenotypes: (1) early-onset mild allergic asthma, (2) early-onset allergic moderate-to-severe remodeled asthma, (3) late-onset non-allergic eosinophilic asthma, and (4) late-onset non-eosinophilic non-allergic asthma [9,10,11] (Figure 1).

Injured epithelial cells synthesize the Thymic Stromal Lymphopoietin (TSLP), high mobility group-box-1 (HMGB1), IL-33 and IL−25 and initiate innate immune responses, highlighting the crosstalk between structural (epithelial cells, fibroblasts, airway smooth muscle cells) and immune cells (mast cells) [21,22,23]. IL-33 and IL-25 activate type 2 innate lymphoid cells (ILC2), while the release of TSLP promotes the activation of dendritic cells (DC) to induce the activation of Th-2 cells and the release of IL-4, IL-5 and IL-13 [5]. IL-4 and IL-13 promote increased mucus secretion, airway hyperresponsiveness, and activation of B cells for IgE isotype production, and IL-5 is critical in activating eosinophilic inflammatory response [24]. High levels of Th-2 inflammatory biomarkers including eosinophils, FeNO, IgE, IL-5 and IL-13, IL-9, prostaglandin D2 (PGD2) are defined as the most common inflammatory Th-2-high endotype of asthma. They can be detected in the bronchial lumen or walls and in the peripheral blood of patients. Indeed, the term eosinophilic asthma is considered a synonym of Th-2-high asthma, encompassing both allergic and non-allergic phenotypes, although with differential response to anti-IL-5/anti-IL-5R antibodies [25]. Th-2-high endotype of airway inflammation drives the transcription of inducible nitric oxide synthase (iNOS), thus increasing NO production in airway epithelial cells, eosinophils, and macrophages. NO is produced through the conversion of the amino acid L-arginine to L-citrulline by the enzyme NOS. The measurement of FeNO is a valuable tool to describe the levels of oxidative stress induced by IL-4 and IL-13 activity, eosinophilic inflammation and steroid responsiveness in the airways. Generally, these biomarkers give an indication of different but at the same time complementary inflammatory pathways and predict disease activity [5,25,26,27]. Higher levels of biomarkers indicate an increased risk for disease exacerbations. Serum IgE implicated in this pathway is correlated with the allergen and with allergic asthma [28]. However, Nolasco et al. highlight the importance of measuring airway mediators rather than the systemic markers to guide asthma management strategy [29].

Eicosanoids are active lipidic mediators involved in the pathophysiology of allergic and non-allergic asthma exerting pro- and anti-inflammatory actions [30]. The high concentrations of urinary LTE4 and PGD2 contribute to identifying adult and childhood severe asthma characterized by type 2 inflammation [31]. Among the urinary eicosanoids, 8-iso-prostaglandin F2a (8-iso-PGF2a), it is a potential biomarker of oxidative stress in asthma. The levels of 8-iso-PGF2a in serum, urine, and sputum are often associated with airway inflammation and airway hyperresponsiveness with a different impact in asthma phenotypes [32]. It is a biomarker of oxidative stress that generates inflammation and remodeling in patients with severe asthma (non-eosinophilic asthma) representing a potential therapeutic target for the treatment of asthma phenotypes [33].

Emerging evidence suggests that treatment response may also be influenced by factors such as obesity, air pollution exposure, and epigenetic mechanisms, which could provide additional important insights into therapeutic variability and disease modulation.

## 3. Asthma and Obesity

**Figure 2:**
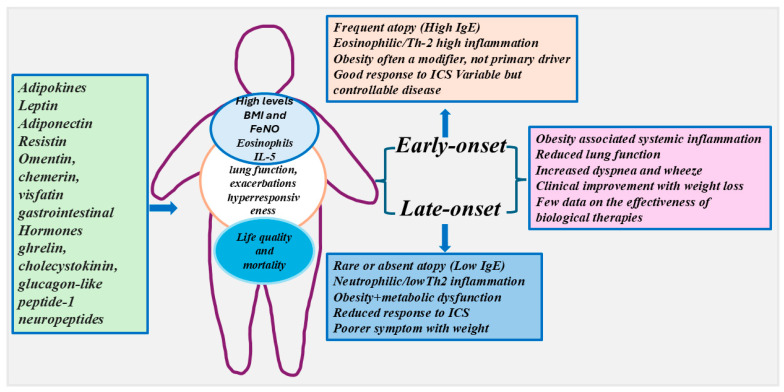
Asthma obesity is associated with both eosinophilic and neutrophilic asthma. Many peptides, such as adipokines, leptin, adiponectin, resistin, omentin, chemerin, and visfatin, gastrointestinal hormones, such as ghrelin, cholecystokinin, and glucagon-like peptide-1, and neuropeptides, such as substance P or neuropeptide Y, are involved in the phenotype of asthma obesity. They contribute to the origin of symptoms and are often implicated in the reduction in lung function, in the increase in exacerbation and airway hyperresponsiveness affecting life quality and mortality of subjects with asthma and obesity. There are two phenotypes of asthma. The first endotype is characterized by an early onset of asthmatic disease with complications due to obesity. It is characterized by high levels of serum IgE, generally associated with allergic processes, and with manifestations of symptoms more severe than in non-obese asthmatics. The second phenotype is characterized by a late onset of asthma due to the obesity of the individual; it shows a high severity of disease in terms of symptoms, low levels of IgE, neutrophilic infiltration and the resistance to corticosteroid therapy. Both early onset and late onset are characterized by obesity associated systemic inflammation, reduced lung function, increased dyspnea and wheeze, clinical improvement with weight loss, and few data showing the effectiveness of biologic therapies. One obese asthma endotype is characterized by an early onset of asthmatic disease with complications due to obesity. This endotype of asthma/obesity has the same prevalence in both sexes and it is characterized by high levels of serum IgE, generally associated with allergic processes, and with manifestations of symptoms more severe than in non-obese asthmatics [37]. The second obese asthma phenotype is characterized by a late onset of asthma due to the obesity of the individual. In this case, the syndrome of asthma/obesity shows a higher severity of disease in terms of symptoms than in lean asthmatic patients with a more severe course of disease, and resistance to corticosteroid therapy. This second phenotype of diseases is not atopic and shows low levels of serum IgE with neutrophil infiltration in the airways [38].

Obesity is a pathological condition representing a risk factor for asthma [34]. The incidence of obesity as a comorbidity of bronchial asthma is constantly increasing worldwide, representing a major health problem [35,36]. Obesity has been associated with airway inflammation and is generally considered a feature of late-onset non-eosinophilic asthma. Severe Asthma Research Program (SARP) studies reported higher levels of IL-5 and eosinophils in the airways of obese individuals with severe asthma, and severe late-onset asthmatic subjects, particularly in females [9,10]. However, obesity can be associated with both eosinophilic and neutrophilic asthma. Many peptides, such as adipokines, leptin, adiponectin, resistin, omentin, chemerin, and visfatin, gastrointestinal hormones, such as ghrelin, cholecystokinin, and glucagon-like peptide-1, and neuropeptides, such as substance P or neuropeptide Y, are involved in the phenotype of asthma obesity [36]. They contribute to the origin of symptoms and are often implicated in the reduction in lung function, in the increase in exacerbations and in airway hyperresponsiveness affecting quality of life and mortality of subjects with asthma and obesity [34] (Figure 2).

One of the principal causes of asthma-related obesity is the release of adipocytokines and proinflammatory mediators. They are responsible for chronic inflammation through the production of reactive oxygen species (ROS) and reduced antioxidant activity [34]. Obese subjects have high free fatty acid levels that trigger endoplasmic reticulum (ER) stress, apoptosis, inflammation, and ROS production increasing the mechanisms of oxidative stress and compromising antioxidant defenses [39]. The markers of oxidative stress are strongly associated with Body Mass Index (BMI), a parameter of obesity in asthmatic subjects [40]. Obese asthmatic patients can possess both type-2 and non-type-2 inflammatory phenotypes [35,41]. The levels of Th-2 biomarkers affect endotypes in patients with asthma obesity, showing implications in the choice of biologic therapy. Higher levels of BMI in asthmatic subjects are associated with higher levels of FeNO and eosinophils, affecting the need for pharmacological treatment. Moreover, it is unclear whether this effect masks the Th2-high state or is due to the development of a true Th-2-low endotype [42,43]. In this scenario, it is important to study the imbalance between oxidative stress and antioxidant activity in subjects with the asthma obesity phenotype, since it actively participates in inflammation by both initiating and sustaining the process [39]. These cellular events lead to metabolic dysfunction and immune dysregulation affecting airway remodeling. The consequences are heightened airway hyperresponsiveness and increased asthma severity, developing a persistent airflow limitation, declining lung function, and a potential increase in asthma-related mortality [34]. However, the intricate mechanisms linking obesity to asthma pathophysiology, particularly concerning airway remodeling, remain insufficiently explored [39,44].

RNA sequencing (RNA-Seq) study conducted on peripheral blood CD4^+^ T cells shows that interferon (IFN)-stimulated genes and IFN-related signaling pathways affect a group of obese asthmatic patients, showing a specific correlation with clinical parameters. These findings provide information on the molecular mechanisms involved in the low type-2 obesity associated asthma phenotype, and promote new stratified therapeutic approaches in asthma obesity [45]. Nevertheless, the interplay between obesity and asthma is far more complex and includes obese tissue-driven inflammatory pathways, mechanical factors, comorbidities, and poor response to corticosteroids. In contrast with the well-defined clinical characteristics of this asthmatic phenotype, the detailed cellular and molecular profiles are not well understood.

Asthmatic subjects with metabolic comorbidities, including cachexia and obesity, may complicate disease phenotypes, leading to increased symptom severity. Recent clinical findings suggest a role for antioxidants and macronutrients in the control of lung function. However, comprehensive translational clinical studies are needed to better understand the role of nutrients in lung function [39]. Evidence supports a possible role of diet in the resilience of chronic inflammatory lung diseases to air pollution exposures. BMI increases the susceptibility of an individual’s respiratory tract to air pollution. A Chinese study showed that higher levels of air pollution have a greater impact on lung function in overweight and obese children [46] and in asthmatic children, with a particularly strong effect in overweight and obese children [40,41,47]. CD4^+^ T-cell genes and pathways involved in IL-7 and IL-32 signaling are differentially expressed in obese children with asthma, which may contribute to their distinct clinical phenotype and steroid resistance. These findings shed insights into the molecular mechanisms underpinning more severe and steroid-resistant asthma among children with obesity [48]. Glucagon-like peptide-1 receptor antagonists are a promising novel pharmacological approach in the treatment of obese asthma airway inflammation. They may have a physiological impact on the excess of fat controlling diaphragm movement or on airway inflammation/adipokine expression [49,50,51]. However, further investigations are needed to identify potential new therapeutic targets for this group of patients, and these observations need to be supported with further epidemiological data to include optimizing translational biomarker discovery and use.

A first systematic analysis of environmental agents of obesity suggests a strong association of air pollution exposure and characteristics of the environment with childhood obesity risk [6,52]. However, it should be emphasized that the results may suffer from bias due to the state of obesity that influences the concentration of biomarkers and therefore the inflammatory state of the airways. In addition, the higher exposure to air pollution is also associated with a higher obesity risk in children, resulting in a vicious circle and accumulation of risk for negative health effects of air pollution [43]. The goal is to identify a population health strategy useful for those with an increased susceptibility to air pollution in the airways such as asthmatic/obese subjects. In addition, further epidemiological data is required to include optimizing translational biomarker discovery and use (Table S1A).

## 4. Asthma and Air Pollution

Air is composed of 78% nitrogen and 21% oxygen (fundamental components) and 1% carbon dioxide and other gases (secondary components). Human activities generate elements from different activities (industrial sources, domestic heating, vehicular traffic), as well as some natural phenomena (e.g., volcanic eruptions) promoting environmental pollution. The substances that modify the composition of atmospheric air are of a different nature. They can be present in both external environments (outdoor) and domestic environments (indoor). The increase in these elements in the air might be a risk factor for respiratory diseases leading to hospitalization and mortality [53]. The need to characterize risk factors associated with the etiology of asthma becomes increasingly urgent for public health. People are not equally susceptible to air pollution, and its effects are different depending on their health status. The asthmatic subject exposed to air pollution presents mucosal edema, mucus production, increased inflammation and constriction of smooth muscle in the airways, with a consequent narrowing of the lumen and an increased collapsibility of the lung depending on the phenotype/endotype and treatments [54]. The susceptible individual shows an alteration in the caliber of the airways with a change in lung function and a reduced airflow limitation. This translates into wheezing, coughing, shortness of breath and/or a sense of chest tightness. The subject exposed to air pollution can have acute or long-term effects associated with altered lung functions in adults and adolescents, but especially in children, with a reduction in forced vital capacity (FVC) (respiratory function of the lower airways) [55,56]. Comorbidities of asthma such as age, specific genetic markers [57,58] or obesity in children can influence in a different manner the effect of air pollution on health [6].

**Figure 3:**
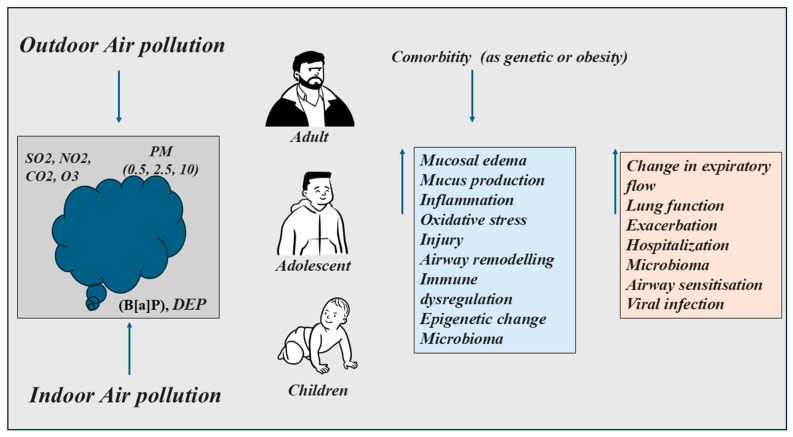
The outdoor/indoor air pollution increases inflammation and symptoms in adults, adolescents and children with asthma, depending on phenotype/endotype. The exposure to air pollution of asthmatic subjects shows an increase in symptoms in the presence of comorbidity.

The exposure of the individual to genetic and environmental factors from birth influences the cellular and molecular dynamics to define the endotype of asthma. Over time more endotypes can contribute to the asthma phenotype [7]. Pollutants trigger epithelial cells to release biomarkers in the airway increasing the risk of subclinical airway inflammation causing respiratory impairment and precluding resolution of inflammation [59]. Recent studies indicate that air pollutants induce asthma through their ability to give rise to oxidative stress, injury, airway remodeling, immune dysregulation, and epigenetic changes in the airway microbiome, enhancing airway sensitization, and viral respiratory tract infections [60]. However, regarding the relationship between air pollutants and asthma risk factors there are many unknowns of great interest for the researchers. Nevertheless, these uncertainties on the topic are a matter of public health concern (Figure 3).

The air contains fine organic or inorganic dust of different dimensions known as particulate matter (PM) for example PM10 and PM2.5 which absorb substances with toxic properties on their surface, such as sulphates, nitrates, metals and volatile compounds [61,62]. The lung is the primary site of PM deposition. PM particles are classified into several size ranges, including PM10 (diameter ≤ 10 μm) PM2.5 (≤2.5 μm) and PM0.5 (≤0.5 μm). Smaller PM move freely in the air, which makes it easier for them to infiltrate the lung, contributing to existing lung problems and exacerbating complications [63]. Inhalation of PM2.5 damages the airways by increasing the inflammatory response and the number of inflammatory cells in the lung. PM2.5 exposure evokes allergic response and airway inflammation, especially in the asthmatic mouse model and leads to Th1/Th2 imbalance. These effects worked mainly by upregulating GATA3 and downregulating Runx3. These data suggest that Runx3 may play an important role in PM2.5-aggravated asthma [64] and disturb the balance of Th17/regulatory cells by targeting glutamate oxaloacetate transaminase 1 and hypoxia-inducible factor 1α in an asthma model [65].

PMs affect the health risks in association with their composition and interactions with other air pollutants [66]. Elevated concentrations of atmospheric PM are associated with an increase in hospital admissions in patients with asthma (subjects at risk) [67]. The direct association between outdoor air pollutants, the risk of asthma and the different phenotypes of asthma is unclear. In China, outdoor air pollution including PM10, PM2.5, sulfur dioxide (SO_2_), nitrogen dioxide (NO_2_), and carbon monoxide (CO) is associated with an increased risk of acute attacks and influences the phenotype of Th-2-high asthma in adolescent males (0–16 years). PM10 and PM2.5 are more harmful to asthma patients with abnormal lung function [68]. The exposure to ozone (O_3_) (an oxidizing three-oxygen gas) and PM10 increased the risk of persistent asthma, and the plasma levels of fluorescent oxidation products (FlOPs) in adult asthmatic subjects [65]. These data suggest a relationship between outdoor air pollution and the mechanisms of oxidative stress in asthma.

NO_2_ causes alterations of oxidative stress mechanisms in generally healthy children and adolescents indicated by high levels of FeNO and reduced respiratory function mimicking the asthma phenotype [55]. NO_2_ exposure is also implicated in the susceptibility of individuals to viral respiratory infections. Elevated NO_2_ concentrations were associated with increased hospital admissions for acute respiratory infections, including viral infection-induced asthma exacerbations, pneumonia and influenza. Short-term environmental exposure to PM increases human susceptibility to viral diseases such as influenza, influenza-like illnesses, RSV bronchiolitis and acute lower respiratory tract infections, including pneumonia [69]. Nhung et al. described a strong correlation between hospitalization for pneumonia and exposure to air pollutants (PM2.5, PM10, SO_2_, O_3_, and NO_2_) in a systematic review and meta-analysis on the effect of ambient air pollution in pediatric asthmatic subjects [70]. Further, the exposure to increased O_3_ was also associated with hospital admission for pneumonia and influenza infection in adults and children with asthma [71,72]. It has been widely demonstrated that human rhinovirus and respiratory syncytial virus are strongly influenced by pollution, but in addition to this, climate change is also a primary causative agent of viral respiratory diseases that can act synergistically on pediatric respiratory diseases, increasing their morbidity and severity [73]. Polycyclic Aromatic Hydrocarbons (PAHs) known as combustion by-products, contribute to the molecular alterations underlying Th-2 or non-Th-2 endotypes of asthma. Childhood exposure to airborne benzo[a]pyrene (B[a]P) is closely related to the clinical diagnosis of asthma, with a greater susceptibility in girls with obesity-associated childhood asthma and in overweight/obese children [74]. Despite these observations it is not clear whether the airborne B[a]P affects atopic asthma in lean children, or if it is robustly associated with non-atopic asthma. B[a]P-asthma associations among the non-atopic children were linked to a profound loss in lung function associated with lower 15-Ft2-isoP and 8-oxodG plasma levels [75]. Exposure to urban traffic-related diesel PM is associated with worse clinical outcomes and unique patterns of inflammation and oxidative stress in children with severe asthma. Children with the highest levels of PM exposure had lower levels of cytokines and T-cells with impaired levels of glutathione and oxidative stress compared to children with lower exposure to diesel exhaust particulate matter (DEP) [76]. DEP contributes to lung decline by promoting inflammation and pulmonary oxidative stress. It increases IL-8 and IL-6 levels while reducing cAMP levels stimulated by forskolin (AC activator), fenoterol (β2-AR agonist) or PGE2 (EP receptor/EP agonist) in airway epithelial cells. In this case, the epithelial response contributes to lung dysfunction induced by air pollution exposure [77]. At the cellular level, DEPs induce oxidative stress, lung inflammation and promote the activation of toll-like receptors by releasing Th-2 and Th-17 cytokines which activate the asthmatic response. Finally, prolonged exposure to pollutants such as DEPs may increase the prevalence of asthma in children, although in adults this causal relationship is less clear [78].

Air pollution present in the indoor sources consists of a unique mixture of contaminants (smoking from tobacco products, cooking practices, heating, combustion of associated fuels, household materials, etc.). Indoor air pollution, due to the enormous amount of time that people spend indoors, is a growing problem for human health, especially for airway health. Higher levels of PM present in indoor air pollution are often associated with an increased negative health consequence for airway symptoms (wheezing, cough, lung function, asthma-related morbidity and mortality, and respiratory infections). The exposure of asthmatic subjects to indoor PM contamination (predominantly PM2.5) is associated with the increased use of rescue medications and/or exacerbations [79,80,81]. The negative impact of PM contamination is concentrated on sensitive and/or vulnerable groups of asthmatic subjects [79]. Recently, it was observed that indoor and outdoor contamination affect the number of exacerbations in patients with severe asthma living in residential areas, and the levels of PM2.5 concentration (mean values of the last 3 years) were significantly correlated with patients showing exacerbations compared with those without. Finally, in this study severe asthmatic subjects with exacerbations showed a high serum level of 8-hydroxy-2′-deoxyguanosine suggesting an increase in oxidative stress in these patients [82].

Numerous scientific and epidemiological studies suggest a high incidence of environmental contamination as a risk factor for asthma in adults and children. Moreover, the high heterogeneity of the reported data highlights the need to identify a standardized methodology to clarify obscure aspects of scientific research in this field. The goal should be to evaluate and overcome the variability in effect estimates that may bias the interpretation of results. Finally, comprehensive measures to reduce the exposure to indoor/outdoor air pollutants are essential to improve asthma outcomes and management in adults and children, in addition to existing guidelines. Various measures can be employed to reduce the effect of environmental exposure in the airways such as the reduction in traffic-related air pollution, increasing green spaces, and reducing exposure to risk factors such as cigarette smoke and allergens in indoor and outdoor places (Table S1B).

## 5. Epigenetic Modifications in Asthma

Major epigenetic mechanisms involve DNA methylation, histone modifications and miRNA. In addition to these canonical mechanisms, increasing evidence highlights a crucial role for epitranscriptomic regulation, particularly RNA modifications such as N6-methyladenosine (m6A), in controlling gene expression in airway diseases including asthma [83,84,85,86]. Many studies demonstrate that environmental pollution has permanent epigenetic effects on gene regulation and expression involving reversible, heritable changes that occur without changing the genetic code [87]. While DNA and chromatin modifications are relatively stable, RNA-based regulation is more dynamic and rapidly responsive to environmental stimuli [84]. Different is for alterations of RNA transcripts since RNAs produced in the nucleus are transcribed in the cytoplasm and subsequently degraded. Accordingly, it might be useful to evaluate the epigenetic changes rather than RNA modifications [88]. However, this view is evolving, as RNA modifications are now recognized as an additional regulatory layer that integrates environmental signals with gene expression programs [83,85].

Epitranscriptomic refers to chemical modifications of RNA molecules that regulate their stability, localization, translation, and degradation without altering the underlying sequence. Among these, N6-methyladenosine (m6A) is the most abundant internal modification in eukaryotic mRNA [83,84]. m6A is dynamically regulated by three classes of proteins: (a) “writers” (methyltransferases), including METTL3, METTL14, and WTAP [83]; (b) “erasers” (demethylases), such as FTO and ALKBH5 [84]; and (c) “readers” (binding proteins), including YTH domain family proteins [85]. These regulators control RNA fate by modulating mRNA splicing, nuclear export, translation efficiency, and decay. Emerging evidence suggests that m6A modification plays a key role in airway inflammation and asthma pathogenesis. Altered expression of m6A regulators has been associated with dysregulated immune responses, including T helper cell differentiation and cytokine production [86,89]. In particular, m6A modifications influence the stability and translation of transcripts encoding inflammatory mediators such as IL-4, IL-5, and IL-13, which are central to type 2 inflammation in asthma [89,90]. Furthermore, m6A has been implicated in epithelial barrier function, airway remodeling, and responses to environmental exposures such as particulate matter [91,92,93]. Changes in m6A patterns may therefore contribute to asthma susceptibility, severity, and treatment response [86]. Importantly, the reversible nature of m6A modifications makes the epitranscriptomic machinery a promising therapeutic target. Pharmacological modulation of m6A “writers” or “erasers” is currently under investigation in inflammatory and respiratory diseases [85,93].

DNA methylation is the addition of a methyl group (CH3) to the 5th carbon atom of the cytosine ring, forming 5-methylcytosine (5-mC), representing 1.5% of genomic DNA. The enzymes DNA methyltransferases (DNMTs) attach a methyl group to DNA leading to gene silencing. When DNA replication occurs, the enzyme DNA helicase separates the double helix into two separate strands, each carrying a methylated cytosine, causing hemi-methylation of the daughter DNA duplex, leaving only one correctly methylated strand. There are three main classes of DNMTs: DNMT1, DNMT3a, and DNMT3b [88].

DNA methylation is important in the pathogenesis of chronic lung disease affecting activation or inactivation of regulatory cell function in response to environmental signals [82,94]. Specific DNA methylations at key CpG sites are biomarkers of lung disease with a predictive function for asthma phenotype including age-related changes in presentation, prevalence, and severity [95]. However, DNA methylation has labile characteristics, and it is difficult to understand whether methylation is a cause or the effect of asthma phenotype [95]. The molecular mechanisms of DNA methylation are largely unknown, and further studies are necessary to describe the modifications of CpG sites in tissues (blood, induced sputum, bronchoalveolar lavage, etc.) of asthmatic subjects.

DNA methylation and histone acetylation are both epigenetic changes observed in the airway epithelium of asthmatic subjects [96]. The pattern of DNA methylation is different in epithelial cells from children with asthma, healthy children or children with allergic asthma [87]. In particular, children with asthma show a differential methylation in the regulatory genes of epithelial barrier function, airway epithelial integrity and immune regulation expressed in airway epithelial cells isolated from bronchial brushing [96]. TSLP and DNA methylation are both risk factors for asthma. The levels of TSLP mRNA and protein increased in airway epithelial cells from asthmatic patients. It was observed that TSLP is a methylation-sensitive gene by the overexpression of DNMT1 in airway epithelial cells of asthmatic subjects [97].

Epigenetic alterations lead to the continuous activation of innate immune cell signals and promote adaptive immune response and epithelial cell activation in chronic inflammation of the lung. The innate immune cellular response causes oxidative stress produced downstream of TLR signaling, induces transient oxidation of guanine bases in the regulatory regions of inflammatory genes causing conformational changes in DNA which in turn trigger inflammation, EMT, and ECM remodeling [98,99]. The NFκB·bromodomain-containing protein 4 (NFκB·BRD4) complex plays a central role in inflammation, triggers mesenchymal transition and extracellular matrix remodeling. During epigenetic alterations, the pleiotropic DNA repair enzyme to 8-oxoguanine DNA glycosylase (OGG1) induces conformational changes in adjacent DNA to recruit the NFκB·BRD4 complex. Targeting epigenetic-inducible OGG1 reduces airway inflammation in preclinical models of asthma [98,99]. Other studies described that DNA methylation at three CpGs (cg05937453, cg25212453 and cg10040131) is often associated with BMI in subjects with different age ranges, adolescent and pediatric. Furthermore, it was observed that DNA methylation correlates with BMI with advancing age. These findings might suggest that differences in DNA methylation are more a consequence rather than a cause of obesity [98,100].

Histone deacetylase (HDACs) and histone acetyltransferase (HATs) play an important role in lung health and pulmonary disease pathogenesis. HDACs, including 11 classical subtypes (HDAC1-HDAC11) and 7 Sirtuins, by their change in the expression and activity, regulate epigenetic mechanisms [101]. HDACs catalyze the removal of acetyl groups from lysine residues of histones and other proteins, generally leading to a closed chromosomal configuration and transcriptional repression. HATs can be classified into two distinct groups (type A and B) on the basis of cellular localization and substrate specificity [102]. In asthmatics, the increase of HAT together with a reduction of HDAC is associated with an increased expression of inflammatory genes [103]. It was observed that cigarette smoking causes a further reduction in HDAC and alters the tight junction expression. The asthmatic subjects who smoke show more severe manifestation of symptoms (phenotype) [103,104,105]. Steroid resistance is a major clinical problem for patients with asthma phenotypes showing neutrophilic inflammation [106]. HDAC2 and IL-17A were correlated with T-helper (Th) 17 cytokines rather than Th2 in severe asthma. Targeting HDAC2 activity as well as T-helper (Th)-17 cytokines is a potential therapeutic approach to reverse steroid insensitivity. The inhibition of HDAC2 activity represents an important therapeutic target to improve epithelial barrier integrity in asthmatic patients [107,108]. Bromodomain and extra terminal (BET) mimics prevent BET proteins from binding to acetylated histones and together with HDAC inhibitors alleviate asthma airway remodeling and hyperresponsiveness and improve asthma symptoms in both mouse models and preclinical experiments [109]. Further studies are necessary to understand the exact roles of specific HDACs in the pathogenesis of asthma and to develop novel and potent HDAC inhibitors showing high selectivity for isoform and BET mimics.

Several studies attempt to describe DNA methylation of different types of cells and tissues of asthmatic patients that regulate airway remodeling, phagocytosis, and other lung functions in asthma [110]. Specific methylation profiles are associated with atopic asthma in the airway epithelium of children, and a nasal methylation panel classifies children by atopy or atopic asthma. These profiles regard CpGs in or near genes relevant to epithelial barrier function (CDHR3 and CDH26), and in other genes related to airway epithelial integrity and immune regulation (FBXL7, NTRK1, and SLC9A3) [111]. Histone methylation is dynamically regulated by histone methylases and demethylases such as LSD1 and JHDM. Post-translational histone modifications play a key role in asthma pathogenesis, and a decrease in H3K27me3 at the promoter of the VEGF gene has been observed in asthmatic airway smooth muscle cells [112]. Histone H3 lysine 18 (H3K18) acetylation increases the expression of EGFR and STAT6 as it is expected to be in the epithelium of asthmatic patients [113]. Epigenetic factors that regulate airway epithelial structure and function are also an attractive area for assessment of susceptibility to asthma. Recent studies on epigenetic regulatory factors have further provided novel insights into the field, particularly their effect on the regulation of some of the asthma susceptibility genes (e.g., methylation of ADAM33). Among the epigenetic regulatory mechanisms, miRNA networks have been shown to regulate a major portion of post-transcriptional gene regulation in the airway epithelium of asthmatic subjects, with some therapeutic potential for miR-19a [113].

Alterations in epigenetic status result in differential gene expressions related to cytokines and transcription factors, resulting in various and distinct phenotypic presentations in asthma. Non-coding RNA fragments known as miRNAs (18–22 nucleotides) and the longer non-coding RNAs (lncRNAs, >200 nucleotides) have an emerging role as regulators of epigenetics and gene transcription either by blocking or by altering mRNA translation stability. They do not transcribe proteins but attenuate transcription and/or limitation according to the guide sequence complementary and play an important role in numerous cellular functions (proliferation, cell differentiation, apoptosis), tissue repair, organ development and immunity of airway diseases, such as asthma phenotypes [114]. Several studies describe the role of miRNAs in the pathogenesis of asthma, reporting data comparing asthmatic patients with controls [115]. Positive correlations have been identified between circulating miRNAs and respiratory functional parameters of patients with allergic asthma. It has been shown that miRNA-let-7 expression inhibits IL-13 expression and cytokine production in asthmatic patients with allergic rhinitis [116]. miRNA-155 and miRNA-126 are involved in allergic airway inflammation and expression levels were correlated with disease progression [117]. Pharmacological reduction of miRNAs promoted reduction in inflammation and eosinophil recruitment, reducing airway hypersensitivity in asthma [118]. Recently a miRNAome-metabolome study demonstrated that 100 significant indirect miRNA-metabolite associations had a collective influence on peripheral blood eosinophilia, AHR, and airflow obstruction in childhood asthma [119]. Results need to be confirmed in the airways of patients with different subtypes of asthma.

Environmental pollution has permanent effects on gene regulation and expression through epigenetic mechanisms. PMs modify target genes contributing to lung pathological conditions such as airway hyperresponsiveness, increased mucus production, airway remodeling (fibrosis, collagen deposition) by DNA methylation, histone modification and miRNA production in asthma [63]. However, although discussing markers as biosensors of disease at the site of lung disease, many studies on the epigenetic effect of pollutants regard epidemiologic studies or data on immune cells of the systemic circulation of rats. The traffic-related PM2.5 exposure aggravated pulmonary inflammation and promoted IFN-γ and IL-4 gene methylation affecting the levels of IFN-γ and IL-4 cytokines production during pregnancy in rats with allergic airway inflammation [120]. The levels of traffic-related PM2.5 affect epigenetic modifications in allergic airway inflammation of rats through impaired regulatory T (Treg) cells function and T-helper type 1 (Th1)/Th2 cells imbalance leading to inflammation exacerbation. These findings show that the framework of epigenetic regulation related to PM2.5 aggravated the allergic airway inflammation in rats [120], and further studies are necessary to confirm these data on asthmatic subjects.

PM2.5 induces epigenetic changes through miRNA production and downstream regulation of asthma-related genes, thereby altering asthma signs and symptoms including exacerbations [63]. Several epigenetic modifications including DNA methylation, histone modification and miRNAs can be used as biomarkers to identify lung complications resulting from PM2.5 exposure. Indoor air pollution, particularly PM_2.5_ and HCHO, aggravates asthma in Chinese children and induces changes in the serum level of miRNA-155 closely associated with the levels in the studied asthma group compared with the control group [121]. Traffic-related PM2.5 enhanced the expression of miRNA-146a and decreased the level of miRNA-31 in rats with allergic airway inflammation leading to exacerbations [103]. Furthermore, recent studies also indicate that pollutants such as PM2.5 can alter epitranscriptomic marks, including m6A methylation, thereby affecting RNA stability and inflammatory signaling pathways [91,92].

DEPs have been tested as genetic mutagens on DNA methylation. The results showed that DEP caused irreversible changes in heterochromatin with effects on the epigenetic state of the human fetus, potentially affecting the lung microbiome and lung functions [122]. However, it is necessary to investigate scientifically the real harmful effects of DEP or of other pollutants on the epigenetic changes involved in asthma exacerbations or in the pathogenesis of disease [78]. Environmental exposures during the prenatal period and early childhood promote the risk of asthma and obesity by generating alterations and epigenetic mechanisms leading to heritable changes often caused by environmental changes and diet [105].

Environmental changes associated with air pollution affected natural systems in terms of biodiversity loss. Exposure to a constantly changing environment due to pollution might be associated with epigenetic alterations. These could provide clues to identify biomarkers related to the onset and progression of airway diseases. The exposome is a concept used to describe environmental exposures that an individual encounters throughout life, and how these exposures impact biology and health [123].

The knowledge about the link between obesity, air pollution and epigenetic modifications in asthma is insufficient. Further studies might be necessary to establish the potential effect of the risk factors on asthma and on response to therapy regarding “obesity, air pollution and epigenetic” (Tables S1C and S2).

## 6. Conclusions

There is a clear need to identify studies and methods to describe how simultaneous exposure to multiple risk factors such as obesity, air pollution and epigenetic alterations may influence the etiology of different asthma phenotypes/endotypes with the aim of better understanding the disease. These goals might be to achieve new and innovative translational clinical studies aimed to identify novel or known biomarkers of disease heterogeneity. In this scenario, the study of epigenetics is of fundamental importance to understand the mechanisms of interaction of risk factors and the immunological response of childhood, adult and elderly asthma.

Finally, we suggest further epidemiological studies that, through an integrated approach, can simultaneously examine the effect of multiple risk factors on clinical and biological parameters of asthma with the aim of promoting the production of One Health data integration approaches. Today these studies are still lacking, and it is hoped that by investing in this area, improvements in phenotyping, diagnosis and treatment of asthma can be achieved.

## Data Availability

No new data were created or analyzed in this study. Data sharing is not applicable to this article.

## Supplementary Materials

The following supporting information can be downloaded at: https://www.mdpi.com/article/10.3390/ijms27104350/s1.
