# Supplementary material for: Obesity, Air Pollution, and Epigenetic Modifications as Risk Factors for Asthma Phenotypes
Source: Int J Mol Sci. 2026 May 13;27(10):4350. doi: 10.3390/ijms27104350 (PMC13207092; doi:10.3390/ijms27104350)
Supplement: Supplementary file 1 [file ijms-27-04350-s001.zip › ijms-4239844-supplementary/Supplementary Table S1.pdf]

Table S1. The major risk factors: A) Obesity; B) Air pollution; C) epigenetic modifications and asthma, underlying mechanisms, key biomarkers and key clinical implications.

A)	Asthma	Main mechanisms	Key biomarkers	Clinical implications
**Obesity**	Early-onset allergic asthma	Th2 inflammation, adipokines, eosinophils	High IgE, eosinophils, FeNO, IL-5	Severe symptoms than non-obese patients
**Obesity**	Late-onset non allergic asthma	Neutrophilic inflammation	Low IgE, neutrophils	Severe disease, reduced effectiveness of corticosteroids
**Obesity**	Metabolic dysfunction-related asthma	Immune imbalance, IFN signaling, T-cell changes	IFN-stimulated genes, eosinophils (variable)	Difficult treatment choice, variable biologic response
**Obesity**	Systemic inflammatory asthma	Oxidative stress, ROS production	Leptin, adiponectin, oxidative stress markers	Reduced lung function, exacerbations
**Obesity**	Metabolic dysfuction-related asthma	Free fatty acids → ER stress, apoptosis	Free fatty acids, ROS, ER stress markers	Reduced lung function, airway remodeling
**Obesity (children)**	Severe pediatric asthma	IL-7 / IL-32 pathways,	IL-7, IL-32	Severe symptoms, steroid resistance
**Obesity + environment**	Pollution sensitive asthma	Increased susceptibility to air pollution	Oxidative stress markers	Reduced lung function, especially in overweight children
**B)**	**Asthma**	**Main mechanisms**	**Key biomarkers**	**Clinical implications**
**PM2.5 / PM10**	Allergic / Th2-high asthma	Airway inflammation, Th2 imbalance, epithelial activation	IL-4, IL-5, IL-13, eosinophils	Reduced lung functions, exacerbations
**PM2.5 / PM10**	Severe / neutrophilic asthma	Oxidative stress, immune dysregulation, airway remodeling	ROS markers, neutrophils	reduced lung function,Steroid resistance
**NO2 / O3 exposure**	"Asthma-like" airway dysfunction	Oxidative stress, reduced lung function	FeNO, oxidative stress markers	Reduced lung functions, Wheezing, cough, exacerbations
**PM2.5 / PM10 + viral infection**	Infection-triggered asthma	Increased viral susceptibility, inflammation	IL-6, IL-8	Hospital admissions, severe attacks, exacerbations
**DEP**	Severe pediatric asthma	TLR activation, oxidative stress, cytokine release (Th2/Th17)	IL-6, IL-8, T-cell changes, glutathione ↓	Worse severity, Decline of lung function
**PAHs (e.g. B[a]P)**	Atopic & non-atopic asthma (children)	Oxidative stress, epigenetic changes	8-oxodG, F2-isoP	Reduced lung function, higher asthma risk
**Indoor PM2.5 exposure**	Allergic / Th2-high asthma	Chronic airway irritation, inflammation	8-OHdG, oxidative stress markers	Exacerbations, wheezing, cough
**C)**	**Asthma**	**Main mechanisms**	**Key biomarkers**	**Clinical implications**
**DNA methylation**	Allergic / atopic asthma	Gene silencing (DNMTs), altered immune regulation, epithelial dysfunction	CpG methylation sites, TSLP, IgE	Disease susceptibility, phenotype prediction
**DNA methylation**	Severe / mixed asthma	Epigenetic regulation of inflammation, airway remodeling	IL-4, IL-5, IFN-related genes	Variable severity, treatment heterogeneity
**Histone modification (HDAC↓ / HAT↑)**	Severe / steroid-resistant asthma	Increased inflammatory gene expression, reduced epithelial integrity	HDAC2, IL-17A	Steroid resistance, worse symptoms

**Histone methylation/acetylation**	Airway remodeling asthma	VEGF, EGFR, STAT6 activation; chromatin changes	H3K27me3, H3K18ac	Airway remodeling, hyperresponsiveness
**miRNA dysregulation**	Allergic / eosinophilic asthma	Post-transcriptional gene regulation, cytokine control	miR-19a, miR-126, miR-155, let-7	Inflammation control, disease progression
**Pollution-induced epigenetics**	Severe / Th2–Th17 imbalance asthma	PM2.5 → DNA methylation, miRNA changes, oxidative stress	IFN-γ, IL-4 methylation, miRNA-155, miRNA-146a	Reduced lung function, exacerbations
**Early-life epigenetic exposure**	Pediatric asthma risk phenotype	Prenatal/early-life epigenetic programming	Epigenetic signatures (CpGs, miRNAs)	Increased asthma risk, long-term effects
