# Supplementary material for: Obesity, Air Pollution, and Epigenetic Modifications as Risk Factors for Asthma Phenotypes
Source: Int J Mol Sci. 2026 May 13;27(10):4350. doi: 10.3390/ijms27104350 (PMC13207092; doi:10.3390/ijms27104350)
Supplement: Supplementary file 1 [file ijms-27-04350-s001.zip › ijms-4239844-supplementary/Supplementary Table S2.pdf]

Table S2. Epigenetic and epitranscriptomic mechanisms in asthma.

**Mechanism**	**Key regulators**	**Molecular function**	**Role in asthma**	**Environmental influence**	**Potential clinical relevance**
**DNA methylation**	DNMT1, DNMT3A, DNMT3B	Addition of methyl groups to CpG sites → gene silencing	Alters immune response, epithelial integrity	PM2.5, smoking, prenatal exposures modify CpG methylation	Biomarkers of asthma risk, disease progression
**Histone modifications**	HATs, HDACs, histone methyltransferases	Chromatin remodeling → transcriptional activation/repression	Increased HAT and reduced HDAC activity → inflammation, steroid resistance	Smoking reduces HDAC activity; pollutants alter chromatin state	Therapeutic targets (HDAC inhibitors, BET inhibitors)
**microRNAs (miRNAs)**	miR-155, miR-126, let-7, miR-146a	Post-transcriptional gene regulation (mRNA degradation/translation inhibition)	Regulate cytokines, eosinophilia, airway inflammation	Pollutants (PM2.5) alter circulating miRNA profiles	Non-invasive biomarkers; therapeutic targets
**Long non-coding RNAs (lncRNAs)**	Various (e.g., MALAT1)	Regulation of transcription and RNA stability	Modulate immune responses and airway remodeling	Environmental stress may alter expression	Emerging biomarkers and therapeutic targets
**Epitranscriptomics (m6A RNA methylation)**	Writers: METTL3, METTL14; Erasers: FTO, ALKBH5; Readers: YTHDF1-3, YTHDC1	mRNA splicing, stability, translation, decay	Controls cytokine expression (IL-4, IL-5, IL-13), Th2 responses, epithelial function	PM2.5 and pollutants alter m6A patterns and regulators	Novel therapeutic targets; regulators of disease severity and treatment response
